# Supplementary material for: Robust Adamantane-Based Membranes with Enhanced Conductivity for Vanadium Flow Battery Application
Source: Polymers (Basel). 2022 Apr 11;14(8):1552. doi: 10.3390/polym14081552 (PMC9029318; doi:10.3390/polym14081552)
Supplement: Supplementary file 1 [file polymers-14-01552-s001.zip › polymers-1632481-supplementary.pdf]

Supplementary Materials

Robust adamantane-based membranes with enhanced conductivity for vanadium flow battery application

Bengui Zhang[1,2*], Xueting Zhang[1], Qian Liu[2], Yanshi Fu[1], Zhirong Yang[1], Haohan Jiang[1], Enlei Zhang[1], Kangjun Wang[1*], Guosheng Wang[1], Zhigang Zhang[1], Shouhai Zhang[2*]

[1] College of Chemical Engineering, Shenyang University of Chemical Technology, Shenyang, 110142, China

[2] State Key Laboratory of Fine Chemicals, School of Chemical Engineering, Dalian University of Technology, Dalian, 116024, China

Materials

Vanadyl sulfate ($VOSO_4$, purity 99%, Shanghai Luyuan Fine Chemical Plant), benzimidazole (98.0%, Shanghai Aladdin Biochemical Technology Co., Ltd.), 1-Methyl-2-pyrrolidinone (NMP), and other reagents were used as received. All other reagents were analytically pure, and they were obtained commercially and used as received.

Nafion 212 membrane was pre-treated by immersing in hot deionized water (80 ^{o}C) for 1 h, and the membrane was immersed in 3 wt. % H_2O_2 solution at 80 °C for 1 h. After that, the membrane was soaked in 1 M H_2SO_4 at 80 °C for 1 h, then washed with deionized water and stored in deionized water.

Synthesis of CAPEK

Chloromethylated adamantane containing poly(aryl ether ketone) (CAPEK) was provided as follows [1]. Adamantane containing poly(aryl ether ketone) (10.0 g) had been dissolved in nitrobenzene (500 mL), then chloromethyl ethyl ether (CMEE) (6 mL) and 2 mL $SnCl_4$ were added to the polymer solution. The temperature of the polymer solution was maintained at 25 ^{o}C for 8 h. After this, the solution was poured into ethanol (500 mL) to precipitate the polymer. To remove all the nitrobenzene, the resulting CAPEK was repeatedly reflux extracted for 12 h using a Soxhlet extractor with ethanol as the solvent and then dried under vacuum at 60 °C for 48 h. The degree of chloromethylation (DC) was then determined using ^{1}H NMR[2], and CAPEK with DC= 0.5 was prepared.

Characterization Methods

* Corresponding author. Tel. :+86 024 89386239; E-mail: zhangbgsc@syuct.edu.cn (B. Zhang); wangkj_dut@syuct.edu.cn (K. Wang); zhangshh@dlut.edu.cn (S. Zhang)

^{1}H NMR spectra of the CAPEK polymer were obtained using a Bruker Avance 500 M and $CDCl_3$ as the test solvent.

FTIR spectra of the membranes were obtained using a Varian 6400 FT-IR spectrometer (32 scans, 4 cm^{-1} resolution, wavenumber range 4000-500 cm^{-1}).

Acid doping level (ADL) and swelling ratio of membranes were tested according to method[3]. ADL is defined as the molar ratio of acid to PAPEK repeat unit in each doped membrane. PAPEK membranes were immersed in H_3PO_4 (85%) for 4 h at different temperatures (60 °C, 90 °C, and 130 °C). Then, the membrane was wiped with filter papers and dried at 80 °C until constant weight, and the ADL was determined with equation (1):

$$\mathrm{ADL}=\frac{W_{acid}-W_{dry}}{W_{dry}}\times\frac{M_{\mathrm{polymer}}}{M_{acid}} \qquad (1)$$

where W_{dry} and W_{acid} are the weights of the PAPEK membrane before and after the doping experiment, respectively. M_{acid} and $M_{polymer}$ are the molecular weights of H_3PO_4 and repeat units of PAPEK, respectively.

The ion exchange capacity (IEC) test of membrane. PAPEK-90 membrane samples were soaked in 1M HCl (200mL) for 24 h to exchange the $HSO4^-$ form into the Cl^- form, then the membrane samples were washed with deionized water and soaked in 1000 mL of deionized water for 24 h to remove residual HCl. Then the Cl-type membrane samples were dried in a vacuum oven at 60 °C for 24h, and weighed as *M*. And then, PAPEK membrane samples in Cl^- form were measured with titration method using 0.05 M $AgNO_3$ and a potentiometric titrator (ZD-2, Shanghai INESA & Scientific Instrument Co., Ltd, China).

IEC was determined using the following equation:

$$\mathrm{IEC\ (mmol\ g^{-1})}=\frac{V_{AgNO3}\times 0.05}{M}\times 100\%$$

where M is the mass of the dried membrane sample in chloride form, V_{AgNO3} is the used volume of $AgNO_3$ solution.

The hydroxyethylpiperazine content of PAPEK membrane. One unit of protonated piperazine group will bind to 2 units of Cl ions. Therefore, the piperazine content of the PAPEK membrane is half the ion exchange capacity (IEC) of the PAPEK membrane (Cl form), which is calculated as IEC /2.

The swelling ratio of PAPEK membranes. The PAPEK membrane samples were dried in a vacuum oven (60°C, 24 hours) and cut into membrane samples with a length and width of 76mm×26mm, and the area (A_{dry}) was calculated. Next, after the H_3PO_4 selective swelling process, filter paper was used to remove residual acid from the membrane samples, then the length and width of the membrane were measured with a Vernier caliper (0.01mm) and the area (A_{acid}) was calculated. The length and width measurement errors were lower than 0.13%

and 0.38%, respectively. The swelling ratio of doped membranes[4] was calculated with equation (2).

$$S_{area} = \frac{A_{acid} - A_{dry}}{A_{dry}} \qquad (2)$$

A_{dry} is the area value of the dry membrane before membrane doping. A_{acid} is the area value of the membrane after membrane doping.

Acid doping level (ADL) and swelling ratio of PAPEK membranes after H_3PO_4 pre-swelling process then immersed in 3 M H_2SO_4 were tested. ADL_{H2SO4} is defined as the molar ratio of H_2SO_4 to PAPEK repeat unit in each doped membrane. PAPEK membranes after H_3PO_4 pre-swelling process were then immersed in 3M H_2SO_4 for 24 h at room temperature. Then, the membrane was wiped with filter papers and dried at 80 °C until constant weight, and the ADL_{H2SO4} was determined with equation (3):

$$\mathrm{ADL}_{\mathrm{H2SO4}} = \frac{W_{H2SO4} - W_{dry}}{W_{dry}} \times \frac{M_{\mathrm{polymer}}}{M_{H2SO4}} \qquad (3)$$

where W_{dry} and W_{H2SO4} are the weights of the PAPEK membrane before and after the doping experiment, respectively. M_{H2SO4} and M_{polymer} are the molecular weights of H_2SO_4 and repeat units of PAPEK, respectively.

The swelling ratio of PAPEK membranes after the H_3PO_4 pre-swelling process then immersed in 3 M H_2SO_4 was calculated with equation (4).

$$S_{H2SO4} = \frac{A_{H2SO4} - A_{dry}}{A_{dry}} \qquad (4)$$

A_{dry} is the area value of the dry membrane before membrane doping in acid. A_{H2SO4} is the area value of the membrane after H_3PO_4 pre-swelling process, then immersed in 3 M H_2SO_4.

Area resistance (R) was determined using AC impedance technique (100 kHz to 20 Hz, AC amplitude 10 mV) (Wayne Kerr 4310, UK). The resistance test cell has two half-cells which were separated with the membrane (effective area, 0.636 cm^2) and filled with vanadium electrolyte (1.5 M $VOSO_4$ in 3 M H_2SO_4). Area resistance was calculated using the following equation (5):

$$R\ (\Omega\ \mathrm{cm}^2) = (R_1 - R_0) \times 0.636 \qquad (5)$$

where R_1 is the resistance of test cell with membrane, R_0 is the resistance of test cell without membrane. The conductivity (δ) was calculated using the equation:

$$\delta = L/R$$

where L was the thickness of membrane and R was the area resistance of the membrane in 1.5 M $VOSO_4$ in 3 M H_2SO_4 , respectively.

Membrane mechanical strength testing. Membranes were measured using a mechanical testing instrument with a standard test method for tensile properties of thin plastic sheeting ASTM D 882-02. The testing speed was 5 mm/min, and the initial gage length was 50 mm at

atmosphere conditions (room temperature, 50% relative humidity). For each membrane, five membrane samples (10 mm × 100mm, 40-45 um thickness) were tested, and their average value was calculated.

Permeability measurements were performed with diffusion cells. The diffusion cell had two half-cells, and the membrane was sandwiched between two half-cells (effective membrane area, 7.068 cm^2), one half-cell filled with 30 mL vanadium solution (1.5 M $VOSO_4$ in 3 M H_2SO_4), and the other half-cell filled with 30 mL $MgSO_4$ solution (1.5 M $MgSO_4$ in 3 M H_2SO_4). The absorbance value of VO^{2+} in $MgSO_4$ solution was determined periodically with a UV-VIS spectrophotometer at 764 nm. Permeability was calculated with the equation (6):

$$V_B \frac{dC_B(t)}{dt} = S\frac{P}{L}(C_A - C_B(t)) \qquad (6)$$

where V_B is the volume of $MgSO_4$ solution; t is the test time, S is the effective membrane area; L is the thickness of membrane; P is the permeability of VO^{2+}; C_A is the concentration of VO^{2+} in vanadium solution; C_B was the concentration of VO^{2+} in $MgSO_4$ solution.

Small angle X-ray scattering (SAXS) testing of membranes. Membrane samples were soaked in water for 24h before the SAXS test. SAXS patterns of the membranes were conducted on a Bruker D8 Advance high resolution diffractmeter between 0.05° and 10°. The scattering vector was calculated according to equation (7):

$$q = \frac{4\pi \sin\theta}{\lambda} \qquad (7)$$

where 2θ is the scattering angle and λ is the scattering wavelength, 0.1541 nm.

The Bragg spacing was calculated as following equation (8):

$$d = \frac{2\pi}{q} \qquad (8)$$

VRFB cell test

VRFB performance measurements were performed using previously reported method[5]. The experimental membranes were sandwiched between two carbon felt electrodes (effective area 5 cm^2, pre-treated in a muffle furnace at 500 °C in the air for four h before use), and this sandwich was clamped between two graphite plates to form a VRFB. The electrolytes for the experimental VRFBs were 20 mL 1.5 M V^{2+}/V^{3+} in 3.0 M H_2SO_4 and 20 mL 1.5 M VO^{2+}/VO_2^+ in 3.0 M H_2SO_4. The negative electrolyte and positive electrolyte were pumped through VRFB. Battery tests were conducted at 25°C using a Neware 5V/3A battery test system. The charge cut-off voltage and discharge cut-off voltage were 1.65 V and 0.8 V, respectively. Cycling tests of VRFB were obtained at a constant current density of 160 mA cm^{-2} and 200 mA cm^{-2}. Due to the vanadium ion imbalance between the positive and negative electrolytes after extended VRFB cycling, the electrolyte in the cell was replaced with a new electrolyte after each 100 cycle sequence.

The DS of CAPEK was defined as the number of chloromethyl groups per polymer repeating unit. ^1H NMR analysis was used to calculate the degree of substitution (DS) of CAPEK. In the ^1H NMR spectrum of CAPEK, the methylene proton in the chloromethyl group appeared at a new peak (4.64 ppm). Therefore, DS is estimated from the integral ratio of 4.64 ppm of -CH_2Cl (H5) to the intact protons (H4, 7.79 ppm) that do not react during the chloromethylation reaction [1,6,7]. The DS of CAPEK was calculated according to the equation: [$DS=2A_5 / A_4$],

where A_5 is the intensity value of the H-5 protons (4.64 ppm), and A_4 is the intensity value of the H-4 protons (7.79 ppm) (Figure S1).

^{1}H NMR for CAPEK, Figure S1. (500 MHz, $CDCl_3$), δ= ppm: 7.90-7.60 (4H, ortho $C_6H_2COC_6H_2$), 7.5-7.3 (4H, $C_6H_2(C_{10}H_{14})C_6H_2$), 7.10-6.85 (8H, $2C_6H_2OC_6H_2$), 4.64 (CH_2Cl), 3.45-1.55 (14H, $C_{10}H_{14}$).

^{1}H NMR for APEK, Figure S2. (500 MHz, $CDCl_3$), δ= ppm: 7.90-7.60 (4H, ortho $C_6H_2COC_6H_2$), 7.5-7.3 (4H, $C_6H_2(C_{10}H_{14})C_6H_2$), 7.10-6.85 (8H, $2C_6H_2OC_6H_2$), 3.45-1.55 (14H, $C_{10}H_{14}$).

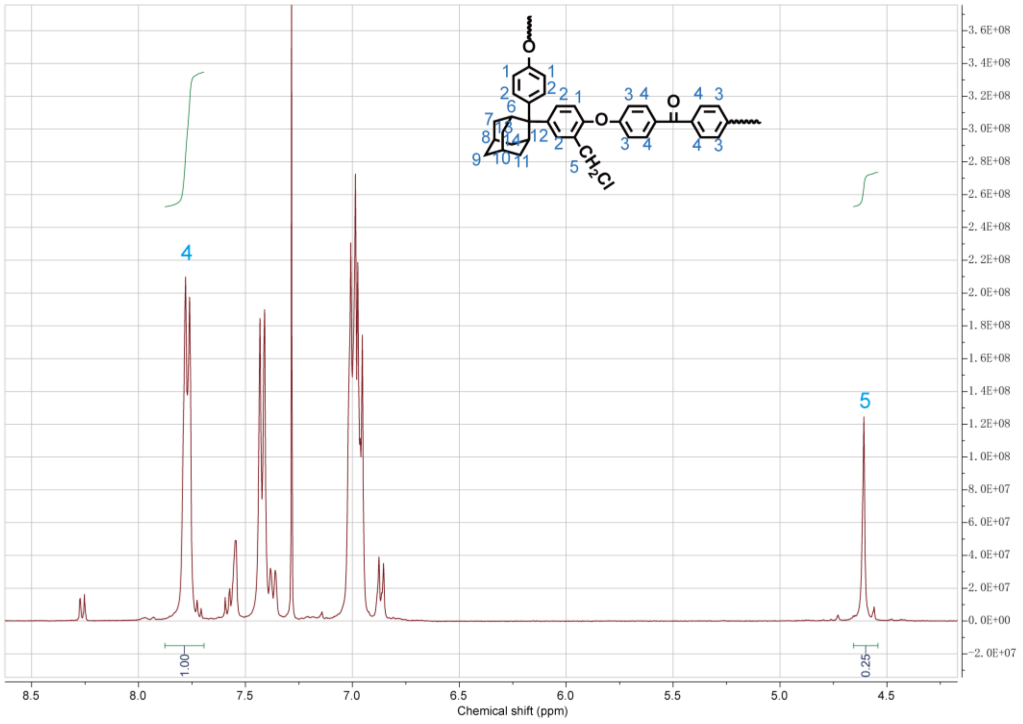

Figure S1. ^{1}H NMR of CAPEK

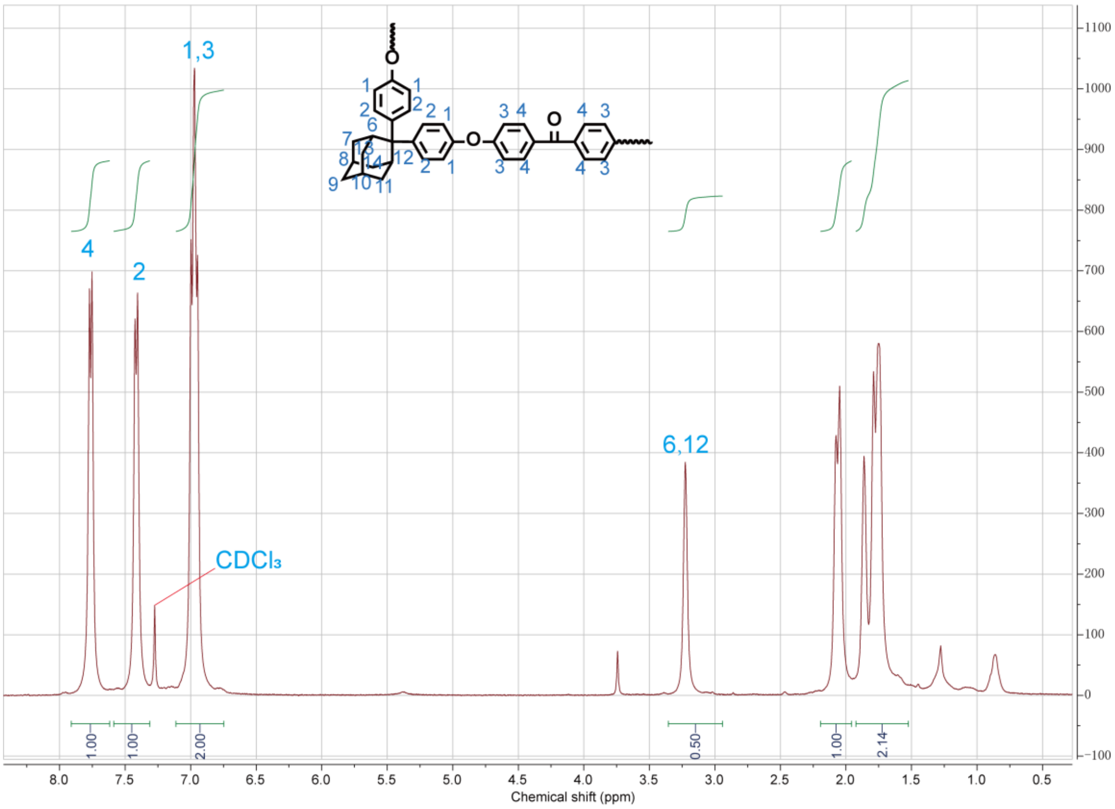

Figure S2. ^{1}H NMR of APEK

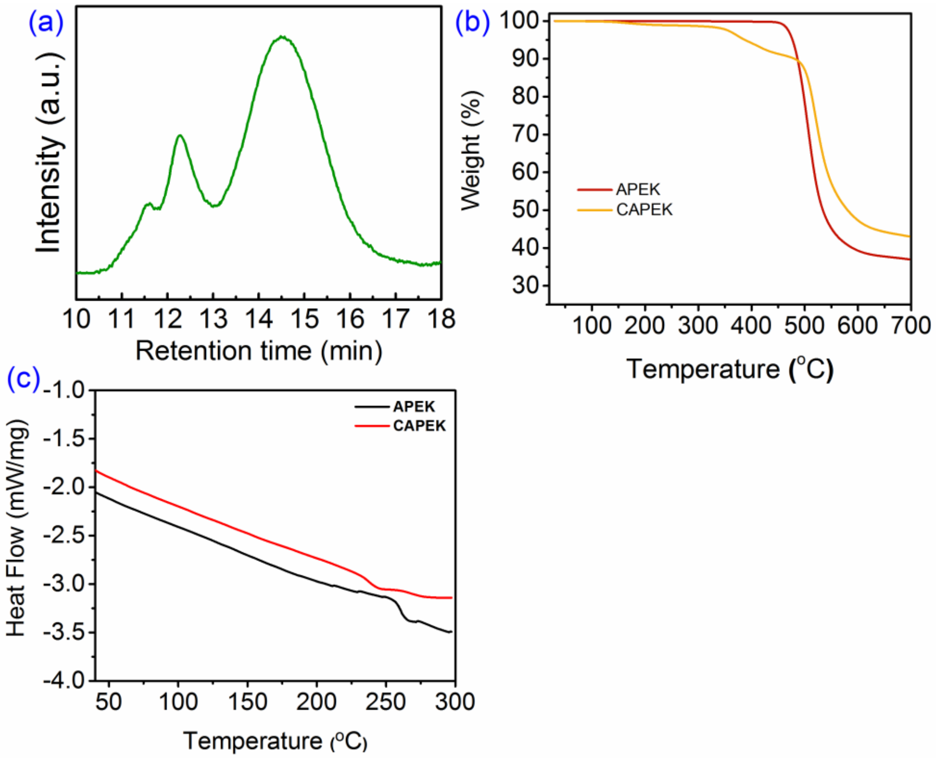

Figure S3 (a) GPC of APEK [8]; (b) TGA of APEK and CAPEK [1] ;(c) DSC of APEK and CAPEK [1]

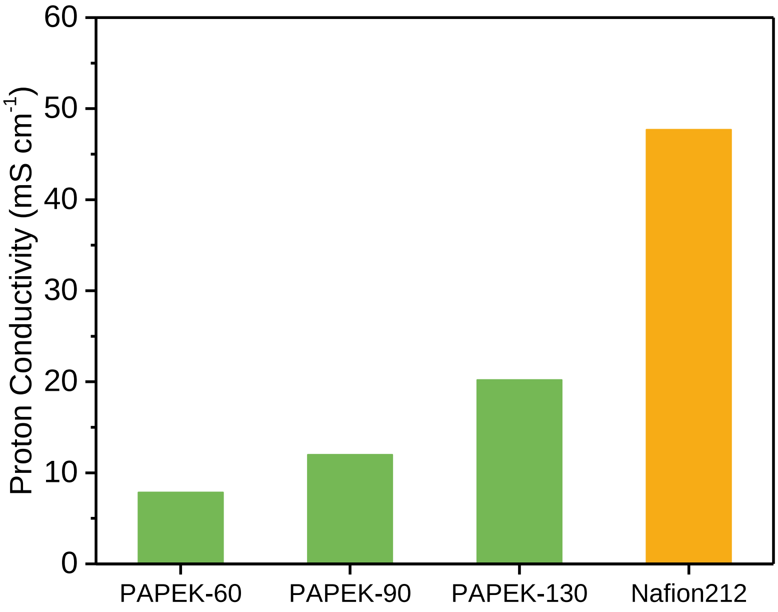

Figure S4 Conductivity of PAPEK and Nafion212 membranes in 1.5M VO^{2+} in 3M H_2SO_4

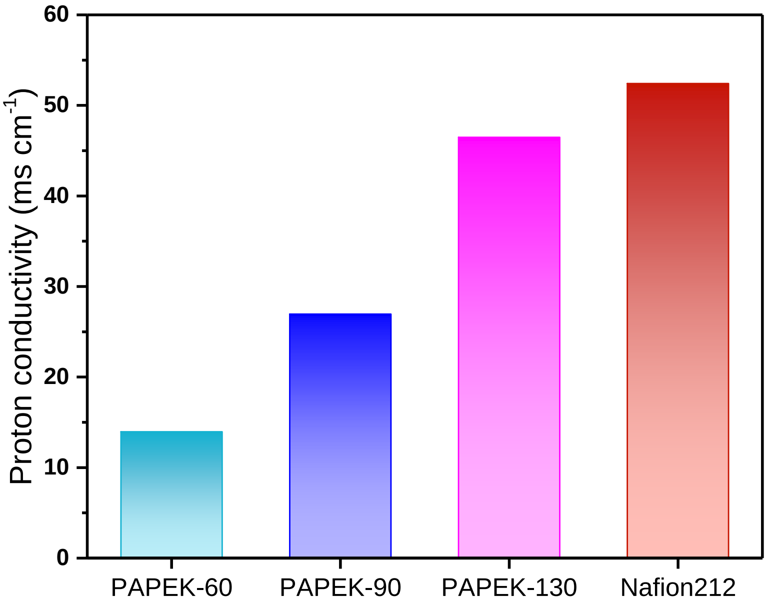

Figure S5 Proton conductivity of PAPEK and Nafion212 membranes in 3M H_2SO_4

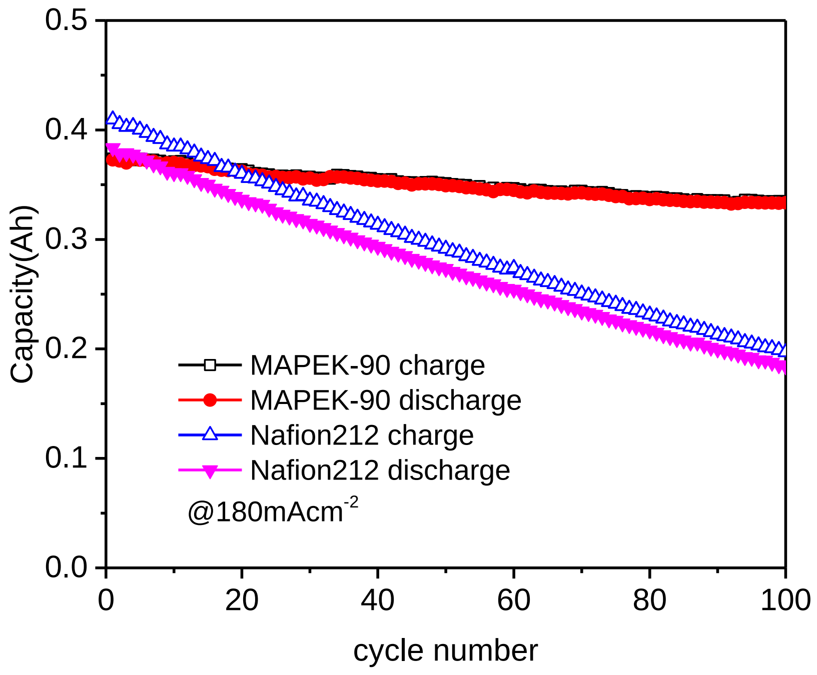

Figure S6 Charge-discharge capacities of VFB with PAPEK-90 and Nafion212 membranes

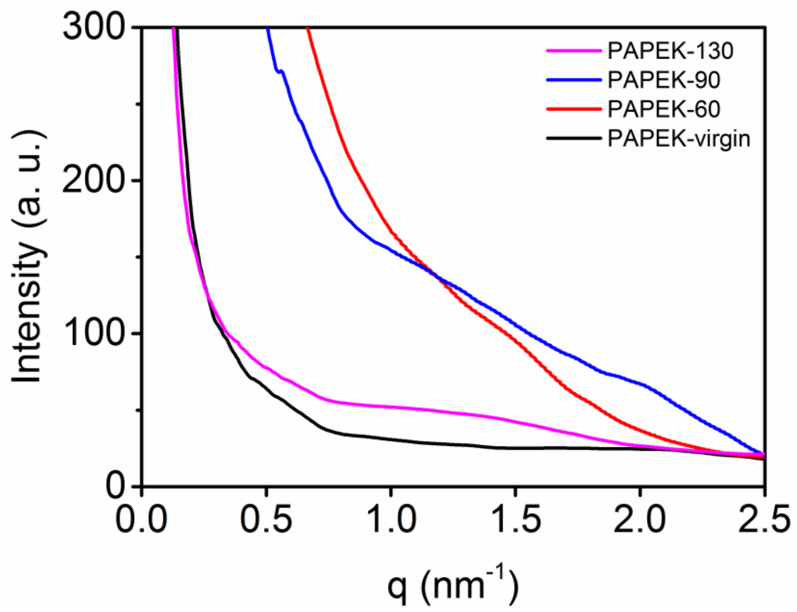

Figure S7 SAXS of PAPEK membranes

Reference

1. Zhang, B.; Wang, Q.; Guan, S.; Weng, Z.; Zhang, E.; Wang, G.; Zhang, Z.; Hu, J.; Zhang, S. High performance membranes based on new 2-adamantane containing poly(aryl ether ketone) for vanadium redox flow battery applications. *Journal of Power Sources* **2018**, *399*, 18-25, doi:10.1016/j.jpowsour.2018.07.050.

2. Zhang, B.; Zhang, S.; Weng, Z.; Wang, G.; Zhang, E.; Yu, P.; Chen, X.; Wang, X. Quaternized adamantane-containing poly(aryl ether ketone) anion exchange membranes for vanadium redox flow battery applications. *Journal of Power Sources* **2016**, *325*, 801-807, doi:10.1016/j.jpowsour.2016.06.101.
3. Peng, S.; Yan, X.; Zhang, D.; Wu, X.; Luo, Y.; He, G. A H3PO4 preswelling strategy to enhance the proton conductivity of a H2SO4-doped polybenzimidazole membrane for vanadium flow batteries. *RSC Advances* **2016**, *6*, 23479-23488, doi:10.1039/c6ra00831c.
4. Peng, S.; Wu, X.; Yan, X.; Gao, L.; Zhu, Y.; Zhang, D.; Li, J.; Wang, Q.; He, G. Polybenzimidazole membranes with nanophase-separated structure induced by non-ionic hydrophilic side chains for vanadium flow batteries. *Journal of Materials Chemistry A* **2018**, *6*, 3895-3905, doi:10.1039/c7ta08790j.
5. Zhang, B.; Wang, F.; Guan, S.; Zhao, M.; Zhang, E.; Wang, G.; Zhang, Z.; Liu, X.; Zhang, S. Pyridinium functionalized, 2-adamantane containing poly(aryl ether ketone) membranes for use in vanadium redox flow batteries. *Journal of Power Sources* **2020**, *477*, 229011, doi:10.1016/j.jpowsour.2020.229011.
6. Zhang, B.; Zhang, S.; Xing, D.; Han, R.; Yin, C.; Jian, X. Quaternized poly(phthalazinone ether ketone ketone) anion exchange membrane with low permeability of vanadium ions for vanadium redox flow battery application. *Journal of Power Sources* **2012**, *217*, 296-302, doi:10.1016/j.jpowsour.2012.06.027.
7. Hossain, M.A.; Jang, H.; Sutradhar, S.C.; Ha, J.; Yoo, J.; Lee, C.; Lee, S.; Kim, W. Novel hydroxide conducting sulfonium-based anion exchange membrane for alkaline fuel cell applications. *International Journal of Hydrogen Energy* **2016**, *41*, 10458-10465, doi:10.1016/j.ijhydene.2016.01.051.
8. Zhang, B.; Fu, Y.; Liu, Q.; Zhang, X.; Yang, Z.; Jiang, H.; Zhang, E.; Wang, K.; Wang, G.; Zhang, Z.; et al. Steric-hindrance benzimidazole constructed highly conductive and robust membrane for vanadium flow battery. *Journal of Membrane Science* **2022**, *646*, doi:10.1016/j.memsci.2022.120254.
